# iNucs: inter-nucleosome interactions

**DOI:** 10.1093/bioinformatics/btab698

**Published:** 2021-10-08

**Authors:** Mehrdad Oveisi, Manu Shukla, Nogayhan Seymen, Masae Ohno, Yuichi Taniguchi, Sunil Nahata, Remco Loos, Ghulam J Mufti, Robin C Allshire, Stefan Dimitrov, Mohammad M Karimi

**Affiliations:** Comprehensive Cancer Centre, School of Cancer & Pharmaceutical Sciences, Faculty of Life Sciences & Medicine, King’s College London, London SE5 8AF, UK; Wellcome Centre for Cell Biology, Institute of Cell Biology, School of Biological Sciences, University of Edinburgh, Edinburgh EH9 3BF, UK; Comprehensive Cancer Centre, School of Cancer & Pharmaceutical Sciences, Faculty of Life Sciences & Medicine, King’s College London, London SE5 8AF, UK; Institute for Integrated Cell-Material Sciences (iCeMS), Kyoto University, Yoshida-Honmachi, Sakyo-ku, Kyoto 606-8501, Japan; Laboratory for Cell Systems Control, RIKEN Center for Biosystems Dynamics Research, Suita, Osaka 565-0874, Japan; Institute for Integrated Cell-Material Sciences (iCeMS), Kyoto University, Yoshida-Honmachi, Sakyo-ku, Kyoto 606-8501, Japan; Laboratory for Cell Systems Control, RIKEN Center for Biosystems Dynamics Research, Suita, Osaka 565-0874, Japan; Institute for Advanced Biosciences, Inserm U 1209, CNRS UMR 5309, Université Grenoble Alpes, 38000 Grenoble, France; BMS Center for Innovation and Translational Research Europe (CITRE, a Bristol Myers Squibb Company), Pabellón de Italia, 41092 Sevilla, Spain; Comprehensive Cancer Centre, School of Cancer & Pharmaceutical Sciences, Faculty of Life Sciences & Medicine, King’s College London, London SE5 8AF, UK; Wellcome Centre for Cell Biology, Institute of Cell Biology, School of Biological Sciences, University of Edinburgh, Edinburgh EH9 3BF, UK; Institute for Advanced Biosciences, Inserm U 1209, CNRS UMR 5309, Université Grenoble Alpes, 38000 Grenoble, France; Izmir Biomedicine and Genome Center, Dokuz Eylul University Health Campus, Balçova, Izmir 35330, Turkey; Comprehensive Cancer Centre, School of Cancer & Pharmaceutical Sciences, Faculty of Life Sciences & Medicine, King’s College London, London SE5 8AF, UK

## Abstract

**Motivation:**

Deciphering nucleosome–nucleosome interactions is an important step toward mesoscale description of chromatin organization but computational tools to perform such analyses are not publicly available.

**Results:**

We developed iNucs, a user-friendly and efficient Python-based bioinformatics tool to compute and visualize nucleosome-resolved interactions using standard pairs format input generated from pairtools.

**Availabilityand implementation:**

https://github.com/Karimi-Lab/inucs/.

**Supplementary information:**

Supplementary data are available at *Bioinformatics* online.

## 1 Introduction

In eukaryotes, DNA is packaged in the nucleus as a nucleoprotein complex called chromatin. The nucleosome is the fundamental unit of chromatin, around which 147 bp of DNA is wound. The packaging of chromatin in the nucleus is not linear. This results in different levels of 3-D genome folding and interaction. Deciphering genome organization within the nucleus is a multi-scale problem and while higher-order tertiary genome structures have been extensively studied using chromatin conformation capture technologies (e.g. 3C, Hi-C etc.), the secondary structure of chromatin, i.e. nucleosomal interactions, remains elusive. Recently, new genome-wide chromatin conformation approaches have been introduced, allowing interactions at nucleosome level. However, the commonly utilized visualization tools such as HiGlass and Juicebox (Kerpedjiev *et al.*, 2018; Robinson *et al.*, 2018) are limited in their ability to provide nucleosome-resolved interactions due to their underlying bin-based methods that do not take into account the nucleosome positions (Supplementary Fig. S1). Therefore, a dedicated bioinformatics tool which allows analysis and visualization of nucleosome interaction networks at individual loci is needed. In this study, we describe ‘iNucs’, a user-friendly and efficient Python-based tool for computing and visualizing nucleosome-level interaction networks. iNucs is designed to receive ligation junctions in pairs format generated by pairtools (https://pairtools.readthedocs.io/en/latest/) and provide nucleosome resolution view consistent with pairsQC (https://github.com/4dn-dcic/pairsqc) output and 4D nucleosome (4DN)-processed files. This allows iNucs to be integrated as a plugin to 4DN analysis pipeline in the future. In addition, the tool provides division and visualization of interaction pairs based on their orientation providing further spatial information about the conformation of chromatin fiber at genomic loci.

## 2 Materials and methods

**Fig. 1.**
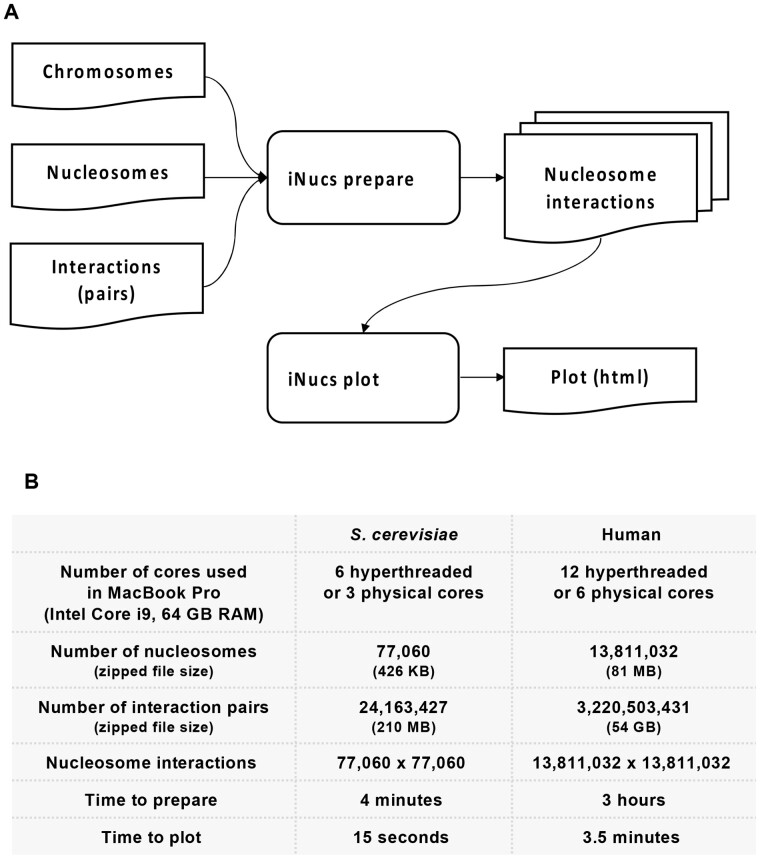
iNUcs overview and performance. (**A**) Schematics of input data requirements, analysis modules and generated output. (**B**) Table showing input data, complexity, computational resource and respective times required to perform the analysis

iNucs consists of prepare and plot modules for which the processes, input and output files and formats are shown in Figure 1A. For a detailed, step-by-step examples of this tool, please see iNucs GitHub page.

*Prepare module:* This module takes in nucleosome genomic coordinates, ligation junctions (in pairs format) and chromosome names as input, and it produces a nucleosome interaction matrix (NIM) as output. The pairs’ input data are typically very large, particularly for human samples (Fig. 1B). Thus, the running time of the underlying algorithms to produce NIM can be potentially very high. To overcome this problem, we reduced the problem of matching DNA interactions with nucleosome coordinates into a sorting algorithm. This in turns allows us to leverage the power of existing sorting algorithms in Python to find an optimized solution for generating NIM. The underlying algorithm in iNucs has a time complexity (*n* log *n*), where *n* is the larger of nucleosome counts and ligation junction counts reported in pairs file (Fig. 1B). One substantial benefit of reducing our problem into sorting is that iNucs can utilize the hardware-supported vectorization capabilities provided by Python libraries NumPy/Pandas that significantly speed up our program. Moreover, iNucs uses advanced parallelization techniques which exploits the power of CPU cores in the system. The detail for optimization of prepare module was explained in iNucs GitHub page.

*Plot module:* This module takes in the NIM from previous module and a query region of interest as input and produces an interactive heatmap plot showing the inter-nucleosome interaction counts within the query region. Here, we focused on the primary aim of the tool, i.e. generation of nucleosome-resolved interaction plots. An important feature of iNucs is that nucleosome-nucleosome interactions are divided in different classes based on ligation orientation of interaction pairs as this information is essential to study spatial arrangement of nucleosomes in the chromatin fiber (Supplementary Fig. S2A and B). Different color scales are used for ease of visualization and differentiation between different classes; purples (plus–minus, inward interactions), greens (minus–plus, outward interactions), reds and blues (plus–plus and minus–minus respectively, both classed as tandem interactions). As both classes of tandem interactions are indistinguishable topologically, they can additionally be represented together in grays. Further, all interactions could be summed up and visualized in oranges or overlaid as individual colors as per investigator’s preference.

iNucs also benefits from an optional feature to compute normalized values for nucleosome-nucleosome interaction counts and plot the original and normalized matrices side by side (Supplementary Fig. S3). We have implemented the Observed/Expected non-zero normalization method described in hicTransform function from HiCExplorer tools (https://hicexplorer.readthedocs.io/en/latest/content/tools/hicTransform.html). The normalized value for each nucleosome pair (NP) is calculated as the observed interaction count for that NP divided by the expected count. The expected count for an NP is the average interaction count between nucleosome pairs whose distance is within the NP's average distance ± a given *distance*. The default value for the parameter *distance* is 200.

To evaluate the performance of iNucs in terms of computational time and memory usage, we applied iNucs on two sets of data for yeast and human (see evaluation and conclusion section), executed on a MacBook Pro (Fig. 1B).

## 3 Evaluation and conclusion

We used recently published *Saccharomyces cerevisiae* Hi-CO (Ohno *et al.*, 2019) and H1 human embryonic stem cell (hESC) line Micro-C (Krietenstein *et al.*, 2020) data to validate iNucs results. The genomic coordinates of nucleosomes for *S.cerevisiae* and ligation junctions (pairs file) were provided by the authors of Hi-CO data. In the case of human data, the pairs file was publicly available in 4DN portal, but there were no matched nucleosome coordinates. Therefore, we downloaded nucleosome coordinates from NucMap repository (https://ngdc.cncb.ac.cn/nucmap/NucMap_FTP_Directory/Homo_sapiens/byDataType/Nucleosome_peaks_DANPOS/) for H1-hESC (Sample ID hsNuc0070101) (Yazdi *et al.*, 2015; Zhao *et al.*, 2019). DANPOS (*Chen et al.*, 2013) was the main tool utilized by NucMap to call nucleosome peaks and coordinates from H1-hESC MNase-seq dataset. We plotted nucleosome-resolved interaction heatmap matrices for specific regions in the respective genomes and compared them with the related heatmaps published in original studies for those regions (Supplementary Fig. S2A and B). As nucleosome resolved heatmap was not available for H1-hESC, we focused on general chromatin organizational features. As expected, local chromatin features such as self-associated domains and boundaries were accurately reproduced by iNucs in the case of H1-hESC (Supplementary Fig. S2A). Also, iNucs generated nucleosome-resolved interaction matrices, which closely resembled nucleosome interaction profiles reported previously for yeast (Ohno *et al.*, 2019) (Supplementary Fig. S2B).

## Supplementary Material

btab698_Supplementary_Data
