# Supplementary material for: iNucs: inter-nucleosome interactions
Source: Bioinformatics. 2021 Oct 8;37(23):4562–3. doi: 10.1093/bioinformatics/btab698 (PMC8652021; doi:10.1093/bioinformatics/btab698)
Supplement: btab698_Supplementary_Data [file btab698_supplementary_data.zip › supplementary_figure1_new.pdf]

Supplementary Figure 1

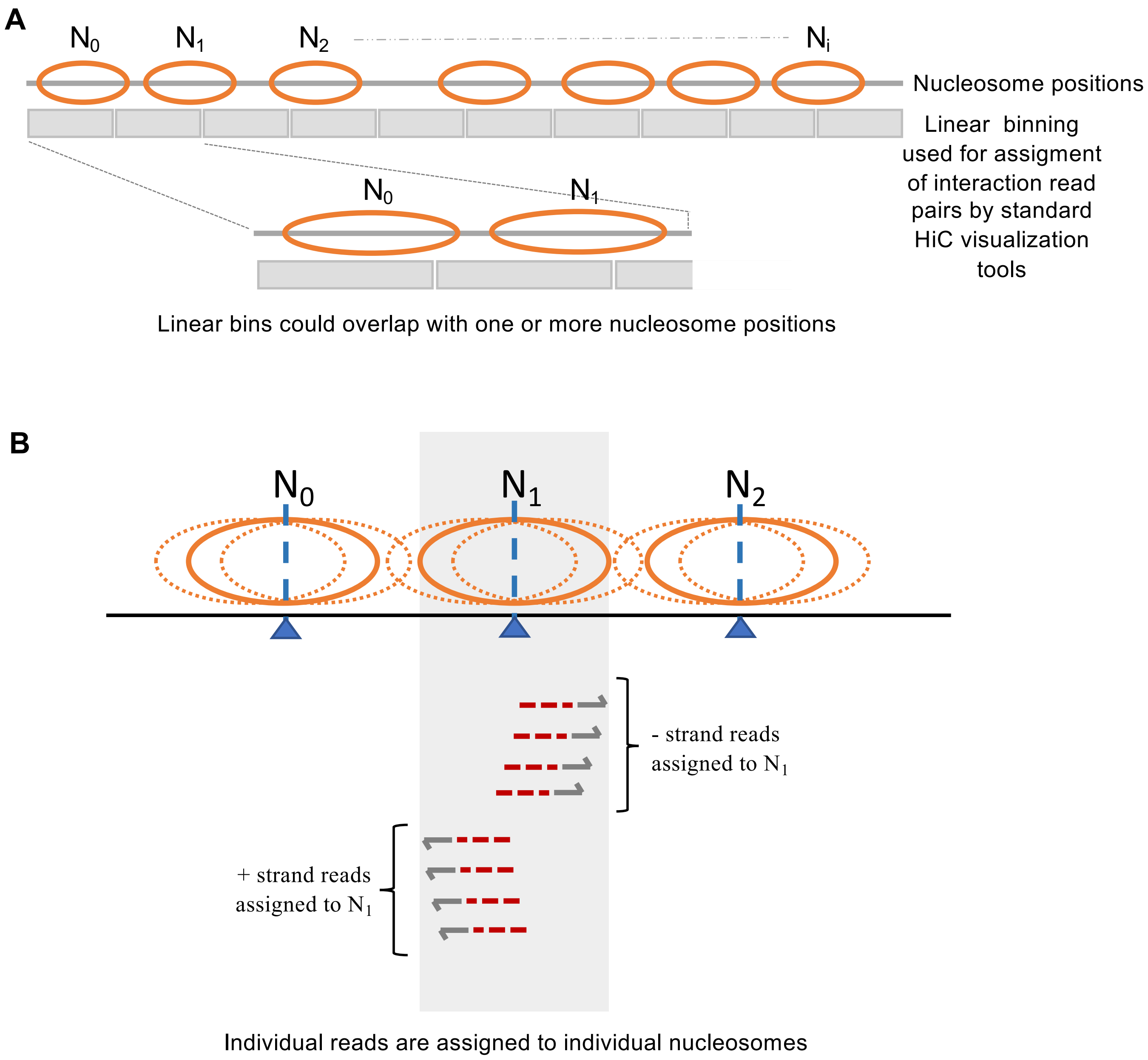

Supplementary Figure 1. (A) Cartoon illustrating the problem in read assignments by standard software when specific nucleosome-nucleosome interactions are of interest. (B) iNucs overcomes this issue by assigning reads to specific nucleosomes guided by their positions in the genome.
