# Supplementary material for: iNucs: inter-nucleosome interactions
Source: Bioinformatics. 2021 Oct 8;37(23):4562–3. doi: 10.1093/bioinformatics/btab698 (PMC8652021; doi:10.1093/bioinformatics/btab698)
Supplement: btab698_Supplementary_Data [file btab698_supplementary_data.zip › supplementary_figure2_new.pdf]

Supplementary Figure 2

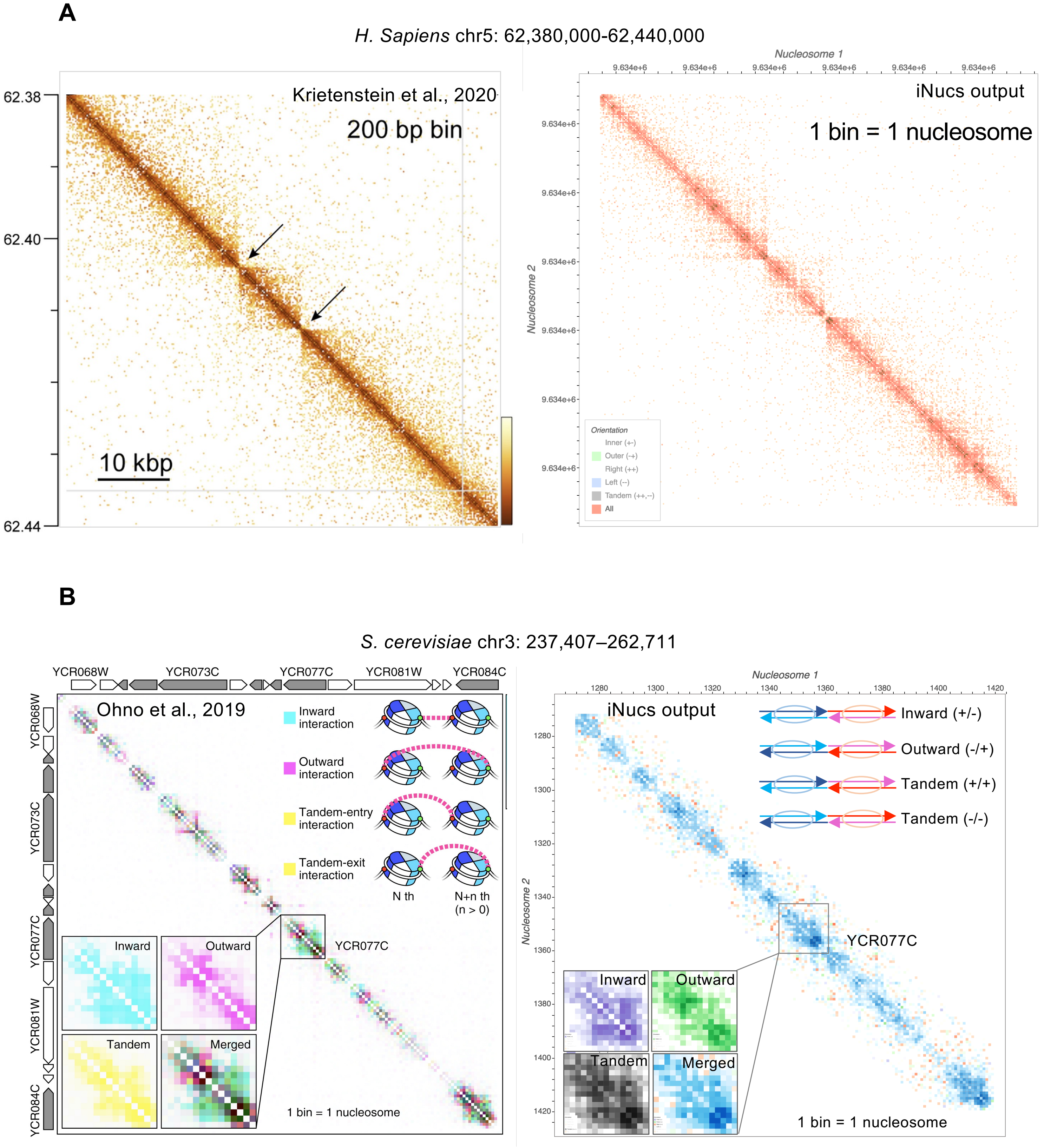

Supplementary Figure 2. Comparison of iNucs heatmaps with previously published heatmaps. Published heatmaps for H1-hESCs and *S. cerevisiae* (Krietenstein et al., Mol. Cell 2020 Fig. 3A and Ohno et al., Cell 2019 Fig. 1B, respectively) are shown in the left panels. (A) While H1-hESCs published heatmap uses linear 200 bp binning, iNUcs employs nucleosome-based binning and splits read pairs based on their orientations. (B) iNucs-generated nucleosome interaction counts in all four orientations (Inward, Outward, Tandem entry and exit) for *S. cerevisiae* are overlaid and compared with a published heatmap in the left panel. Zoomed-in region for YCR077C gene is shown in all possible read pair orientations both individually and merged in insets.
