# Supplementary material for: iNucs: inter-nucleosome interactions
Source: Bioinformatics. 2021 Oct 8;37(23):4562–3. doi: 10.1093/bioinformatics/btab698 (PMC8652021; doi:10.1093/bioinformatics/btab698)
Supplement: btab698_Supplementary_Data [file btab698_supplementary_data.zip › supplementary_figure3_new.pdf]

Supplementary Figure3

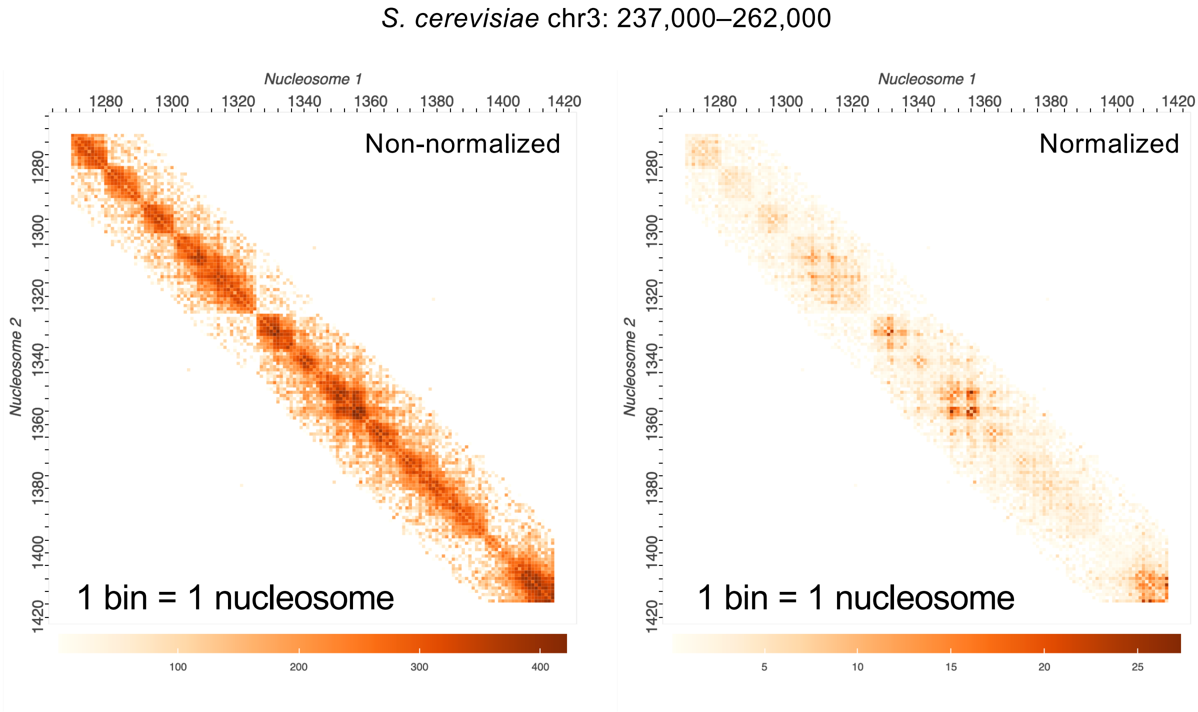

Supplementary Figure 3. Non-normalized and normalized outputs from iNucs. Total nucleosome interaction heatmaps were generated from the Hi-CO data described in Ohno et al., 2019, for the indicated *S. cerevisiae* genomic region (left panel–standard heatmap, right panel–normalized heatmap). Sum of total nucleosomal interactions in all possible orientations (inward, outward and tandem) are shown.
